# LIS1, a glyco-humanized swine polyclonal anti-lymphocyte globulin, as a novel induction treatment in solid organ transplantation

**DOI:** 10.3389/fimmu.2023.1137629

**Published:** 2023-02-16

**Authors:** Juliette Rousse, Pierre-Joseph Royer, Gwénaëlle Evanno, Elsa Lheriteau, Carine Ciron, Apolline Salama, Françoise Shneiker, Roberto Duchi, Andrea Perota, Cesare Galli, Emmanuele Cozzi, Gilles Blancho, Odile Duvaux, Sophie Brouard, Jean-Paul Soulillou, Jean-Marie Bach, Bernard Vanhove

**Affiliations:** ^1^ Research and Development, Xenothera, Nantes, France; ^2^ Nantes Université, Inserm, University Hospital Center CHU Nantes, Center for Research in Transplantation and Translational Immunology, UMR 1064, ITUN, Nantes, France; ^3^ Transplantation Immunology Unit, Padua University Hospital, Padova, Italy; ^4^ Avantea, Laboratorio di Tecnologie della Riproduzione, Cremona, Italy; ^5^ Oniris, INRAE, IECM, USC 1383, Nantes, France

**Keywords:** solid organ transplantation, induction, anti-lymphocyte globulin, anti-lymphocyte antibody, polyclonal antibodies (PAbs), kidney trans plantation, pig IgG

## Abstract

Anti-thymocyte or anti-lymphocyte globulins (ATGs/ALGs) are immunosuppressive drugs used in induction therapies to prevent acute rejection in solid organ transplantation. Because animal-derived, ATGs/ALGs contain highly immunogenic carbohydrate xenoantigens eliciting antibodies that are associated with subclinical inflammatory events, possibly impacting long-term graft survival. Their strong and long-lasting lymphodepleting activity also increases the risk for infections. We investigated here the *in vitro* and *in vivo* activity of LIS1, a glyco-humanized ALG (GH-ALG) produced in pigs knocked out for the two major xeno-antigens αGal and Neu5Gc. It differs from other ATGs/ALGs by its mechanism of action excluding antibody-dependent cell-mediated cytotoxicity and being restricted to complement-mediated cytotoxicity, phagocyte-mediated cytotoxicity, apoptosis and antigen masking, resulting in profound inhibition of T-cell alloreactivity in mixed leucocyte reactions. Preclinical evaluation in non-human primates showed that GH-ALG dramatically reduced CD4^+^ (p=0.0005,***), CD8^+^ effector T cells (p=0.0002,***) or myeloid cells (p=0.0007,***) but not T-reg (p=0.65, ns) or B cells (p=0.65, ns). Compared with rabbit ATG, GH-ALG induced transient depletion (less than one week) of target T cells in the peripheral blood (<100 lymphocytes/L) but was equivalent in preventing allograft rejection in a skin allograft model. The novel therapeutic modality of GH-ALG might present advantages in induction treatment during organ transplantation by shortening the T-cell depletion period while maintaining adequate immunosuppression and reducing immunogenicity.

## Introduction

“Induction therapies” refer to early lymphodepletion or blockade to avoid acute graft rejection in solid organ transplantation. They also reduce nephrotoxicity by delaying the introduction of calcineurin inhibitors which are maintenance agents (1, 2). They comprise CD25 antagonists (3), anti-CD52 antibody and anti-lymphocyte/thymocyte globulins (ALGs/ATGs) (4, 5). ALGs/ATGs are polyclonal antibodies directed against T- and B-lymphocytes (6), obtained from rabbits or horses immunized with primary human thymocytes or human T-cell lines (7, 8). Their activity rely on apoptosis induction (9, 10), complement-dependent cytotoxicity (CDC), antibody-dependent cell cytotoxicity (ADCC), phagocytosis and other nondestructive mechanisms (6).

ALGs/ATGs have been used for 50 years (4, 6) and have proven efficacy in preventing acute graft rejection. However, they are endowed with a strong immunogenicity associated with side effects ranging from mild fever or skin rashes to more serious serum sickness disease (SSD) (11, 12) or anaphylactic shock (13–15). SSD is a hypersensitivity reaction toward glycoproteins from animal sources (16) caused by antibodies directed against glycans bearing the common mammalian sialic acid N-glycolylneuraminic acid (Neu5Gc) (17–21). Humans present a biased sialylation of glycoproteins and glycolipids. The lack of Neu5Gc (22) on proteins or lipids is due to a human lineage-specific genetic mutation in the enzyme cytidine monophosphate-N-acetylneuraminic acid hydroxylase (CMAH) (22). A direct consequence is that Neu5Gc epitopes are excluded from «self-tolerance» and elicit anti-Neu5Gc antibodies in humans (17, 23–26). In the same way, humans also lack the α1,3-galactosyl-transferase enzyme (GGTA1) (27) and are not tolerant to α1,3 galactose (Gal) epitopes; they present various levels of natural anti-a1,3Gal antibodies and increase their level of anti-a1,3Gal antibodies after infusion with animal-derived products (28–30).

Rabbit ATG given to diabetic patients resulted in highly significant increases in anti-Gal and -Neu5Gc IgG/IgM, still detectable one year post-infusion and responsible for the induction of SSD and immune complex disease in almost all patients (25). Even in the context of concomitant administration of immunosuppressants and steroids, however, such as in organ transplantation, SSD still occurs in 10% of recipients who received rabbit ALG/ATG (11). SSD, however, is a contributing factor to late graft loss following ALG/ATG induction (11) and high anti-Neu5Gc antibody level after kidney transplantation has also been associated with late graft loss (11). This effect may be due to the well-known passive incorporation of Neu5Gc residues from food origin into endothelial and epithelial cells (31, 32), possibly resulting in a chronic inflammation called “xenosialitis” in the presence of anti-Neu5Gc antibodies, although the detrimental role of these “natural antibodies” in human still needs to be confirmed (24, 33) ^(^ 34 ^),^. In parallel, anti-Gal antibody responses can be involved in anaphylaxis-type reactions targeting glycoconjugates present on therapeutic products of animal origin (23, 25).

Rabbit ATG is known for its rapid, intense and persistent depletion. Indeed, it is characterized by a depletion of 83-92% of almost all lymphocyte subpopulations, observable on the day following treatment. It also results in leukopenia and thrombocytopenia (6). This depletion usually lasts for up to 3 months and may even extend to 6 months, which increases the risk of infections (35, 36). Anti-CD25 monoclonal antibodies (α chain of the IL-2 receptor) do not cause lymphocyte depletion. Its mechanism is based on blocking the binding of IL-2 to its receptor, which is only expressed by activated cells (37). Clinically, basiliximab has comparable efficacy to ATG in terms of survival at one year and reduces infections, but at the cost of a slightly higher rate of acute rejection (2, 38). Another anti-CD52 monoclonal antibody is also used: alemtuzumab. This causes massive depletion of peripheral blood T cells (CD52 is found on T and B lymphocytes, monocytes, macrophages and granulocytes) (39). However, alemtuzumab is not widely used in the clinic, as it has not yet been the subject of a prospective, randomized study (38).

To address these issues, a “glyco-humanized” polyclonal serum, has been developed in pigs knocked out for the two genes CMAH and GGTA1 so that the two main xenogenic glycoantigens have been changed into human-type glycoantigens: the a1,3Gal epitopes are replaced by Gal β1,4-GlucNac, and Neu5Gc is replaced by Neu5Ac. The mechanisms of action of GH-ALG were investigated *in vitro* on human PBMC and *in vivo* in nonhuman primates. They differ from human rabbit immunoglobulins because they have the unique property of not interacting with human FcγR, which alters the mechanism of action by altering ADCC (40).

## Methods

### GH-ALG manufacturing and composition

Double KO defined high health status pigs were immunized with a human CD4^+^ CD8^+^ T-cell line. GH-ALG, the IgG fraction, was purified from serum in compliance with good manufacturing practice (GMP) and ICH guidelines. Details have been published elsewhere (40).

### Competition assay to determine the GH-ALG target repertoire

Human peripheral blood mononuclear cells (PBMCs) were plated at 1.10^5^ cells per well in 96-well plates and then were labeled with GH-ALG (250 µg/mL) and/or with the following monoclonal antibodies used at a 1/100 dilution: anti-human TCRa/b-FITC (Clone T10B9.1A.31); anti-human CD2-PE (Clone RPA-2.10); anti-human CD3-PE (Clone HIT3a); anti-human CD4-PE (Clone RPA-T4); anti-human CD8-PE (RPA-T8); and anti-human CD28-PE (CD28.2) (all from BD Biosciences, Franklin Lakes, United States). Cell fluorescence was then measured by flow cytometry. A mean fluorescence intensity drop indicated competition between GH-ALG and the test antibody.

### Determination of the GH-ALG active fraction

The active fraction of GH-ALG was determined by serial depletion against the cells used for immunization. Briefly, 30x10^6^ target cells fixed in 4% paraformaldehyde were resuspended in 1 mL of GH-ALG (1.6 mg/ml) in phosphate buffered saline (PBS). After 20 min of incubation at room temperature, the cells were pelleted and the supernatant was transferred to a second tube containing a new fresh cell pellet to repeat the incubation. Five successive incubations were performed. The remaining specific antibodies were monitored after each incubation using a binding assay. The total IgG concentration was evaluated by spectrophotometry. The GH-ALG active fraction was defined as the fraction of IgG cleared using this repeated depletion procedure.

### Binding assay

The binding of GH-ALG to PBMC, red blood cells and platelets from healthy was measured by flow cytometry. Briefly, 10^5^ blood cells were incubated with increasing concentrations of pig or rabbit ALGs/ATGs for 30 min at 4°C. After 3 washes, bound IgG were detected using FITC-conjugated anti-pig or anti-rabbit antibody (both from Bio-Rad) or AF-488 conjugated protein G (Thermo Fisher, Waltham, MA), and analyzed on a BD CELESTA Flow cytometer.

### Comparison of pig and rabbit IgG detection by G protein

#### ELISA

GH-ALG and rabbit-ATG are coated on a maxisorp plate at 50ug/mL. After saturation and washing, revelation is performed with Alexa Fluor 488 conjugated protein G diluted at 1/200 or 1/400 and the reading performed in fluorescence with TECAN.

#### BLItz

To perform the kinetic binding analysis of pig and rabbit IgG to protein G, we use the BLItz technique (VWR, Radnor, Pennsylvanie, United States) using bio-layer interferometry technology. Briefly, a first step of hydration of the G-protein biosensors for 10min is followed by loading the biosensors with GH-ALG or rabbit-ATG at 10ug/mL. Reading with BLItz for 2-5min. Results are interpreted using the affinity constant between IgGs and protein G.

### Apoptosis assay

PBMCs were mixed with increasing doses of GH-ALG or rabbit ATG in RPMI medium with 10% FCS. Nonimmune IgG was used as a negative control. After 3 h of culture (37°C, 5% CO_2_), the cells were labeled with AF488-conjugated Annexin V and DAPI (Thermo Fisher) before analysis by flow cytometry. The percentages of cells in early apoptosis (Annexin V+/DAPI- cells) and late apoptosis (Annexin V+/DAPI+) were combined to determine the overall % of apoptosis.

### Platelet aggregation assay

Human platelets purified from citrated blood were mixed with increasing doses of GH-ALG or rabbit ATG and aggregation was monitored for 60 minutes on a TA-8V STAGO aggregometer. To induce platelet aggregation, ristocetin (1.5 mg/ml), Thrombin Receptor Activator Peptide (TRAP,1.5 µM), arachidonic acid (0.3 mg/ml), ADP (adenosine diphosphate, 5 µM), epinephrine (5 µM) and collagen (1.25 µg/ml) were evaluated.

### Opsonophagocytosis assay

Monocytes were purified from PBMCs by CD14 positive magnetic selection (Miltenyi Biotec, Bergisch Gladbach, Germany) and plated at 2.10^6^ cells/ml in RPMI 1640 medium containing 10% fetal calf serum (FCS). Monocyte differentiation into macrophages was performed for 5-7 days in the presence of human M-CSF (100 ng/ml) (R&D system). On the day of the assay, CFSE-labeled CD3 lymphocytes were incubated with GH-ALG in RPMI medium with 10% heat-inactivated FCS for 30 min at 4°C. Lymphocytes were washed twice with medium and cultured with human macrophages (ratio 1:1). After 3 hours of culture, the cells were washed twice, and macrophages were labeled with CD14-BV421 for 30 min at 4°C, and analysis by flow cytometry. Phagocytosis was assessed as the percentage of double-positive (CFSE^+^/CD14^+^) cells over CD14^+^ cells.

### Mixed lymphocyte reaction assay

Proliferation was assessed using a CFSE dilution assay. Briefly, stimulator PBMCs were irradiated at 35 Gy and cultured (ratio 4:1) with CFSE-labeled responder PBMCs (Invitrogen, Waltham, Massachusetts, United States of America). The cells were cultured for 3 days in RPMI 10% FCS in the presence or absence of GH-ALG or rabbit ATG and analyzed by flow cytometry. Groups were compared using ANOVA and the Tukey−Kramer multiple comparison test. The results were expressed as %FITC low × number of lymphocytes to analyze cell proliferation of the residual cells.

### *In vivo* evaluation

#### Animals

Eighteen cynomolgus monkeys were used after authorization of the French Research Ministry (APAFIS 10717)/Citoxlab France Ethics Committee (CEC)/Animal Welfare Body of Cynbiose/Ethics Committee of VetAgro-Sup. The characteristics of these *in vivo* studies are reported in **Supplementary Table 1**. Cynomolgus monkeys received GH-ALG by daily intravenous administration at doses ranging from 40 to 75 mg/kg/administration for 5 days. GH-ALG was administered as a solution in the vehicle (sterile NaCl 0.9%) under a constant infusion rate. In the rabbit ATG group, 2 macaques were injected 5 times at 5mg/kg intravenously.

#### Lymphocyte depletion

CD3^+^ T lymphocyte numeration was performed using the BD Trucount system (BD Biosciences). Whole blood cells were labeled with the fluorochrome-labeled monoclonal antibody anti-CD3 (BD Biosciences). Other lymphocyte subpopulations were studied by flow cytometry. Briefly, fresh peripheral blood cells were stained with fluorochrome-labeled monoclonal antibodies against CD3 (SP34-2), CD4 (RPA-T4), CD8 (RPA-T8), CD20 (2H7), CD25 (M-A251), CD27 (T27.1), CD28 (CD28.2), CD31 (WM-59), CD45 (D058-1283), CD45-RA (5H9), CD95 (DX2), CD127 (hIL-7R-M21), GzmB, and FoxP3 (236A/E7) (Beckton Dickinson, San Jose, CA, USA). Polychromatic flow cytometric analyses were performed using a Canto II analyzer (BD Biosciences).

#### Pharmacokinetic analysis

The GH-ALG concentration in monkey serum was determined using a pig IgG ELISA (Bethyl, Montgomery, Tx) according to the manufacturer’s instructions.

#### Skin allograft model in cynomolgus monkeys

Skin grafts were performed on 5 anesthetized female cynomolgus monkeys (**Supplemental Table 1**). After back skin shearing and asepsis, the animals were grafted in pairs (back skin collection using a 20 mm diameter template, followed by grafting and skin graft suturing). In the back of each animal, three grafts were performed: one autograft and two pairwise allografts. In each group, one animal received skin transplants of his congener and vice versa. Three of them (Group 1) received GH-ALG at 75 mg/kg once a day for 5 consecutive days. The injection was performed intravenously (concentration: 10 mg/mL; flow rate: 4 mL/kg/h; infusion duration: approximately 1 h50). The two remaining monkeys (Group 2) were used as controls and did not receive treatment.

#### Statistical analysis

All statistical analyses were performed using GraphPad Software (GraphPad Software, San Diego, CA). p values of <0.05 were considered statistically significant.

## Results

### Specificity of target

#### Binding of GH-ALG to human blood cells

**Figure 1:**
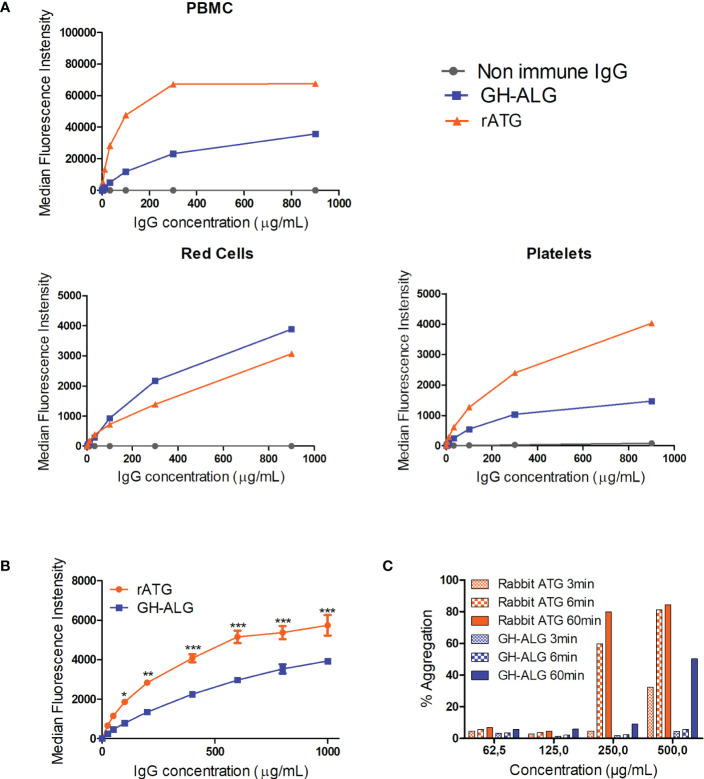
Interaction between GH-ALG and human blood cells. **(A)** Binding of GH-ALG or rabbit ATG to human PBMCs, red cells and platelets was investigated by flow cytometry. Cells were incubated with increasing concentrations of GH-ALG or rabbit ATG. Nonimmune IgG was used as a control. After washing, bound IgG was detected by flow cytometry using a FITC-conjugated anti-pig antibody or FITC-conjugated anti-rabbit antibody (N=3). **(B)** Direct comparison of GH-ALG and rabbit ATG binding to human platelets. Human platelets were incubated with increasing doses of GH-ALG or rabbit ATG. Attached antibodies were then detected using AF488-conjugated protein G and flow cytometry analysis. The data are expressed as means ± SEM (N=3). Two-way ANOVA (*, p<0.5; **, p<0.01; ***, p<0.005). **(C)** Platelet aggregation assays. Human platelets were purified from citrated blood, and aggregation was measured by turbidimetry using a TA-8 V platelet aggregometer (Stago). One representative experiment from two is shown. .

The binding of GH-ALG to human PBMCs, platelets or red blood cells was investigated by flow cytometry and compared with that of rabbit ATG (**Figure 1**). A dose-dependent and saturable signal was obtained for GH-ALG and rabbit ATG and was higher for rabbit ATG with maximum MFI at 40,000 for GH-ALG and 50,000 for thymoglobulin. IgG from nonimmunized pigs was used as a negative control and displayed negligible binding (Mean fluorescence intensity, MFI < 82). GH-ALG binding to red blood cells was very low (MFI < 5000; **Figure 1A**) for GH-ALG and rabbit ATG. Binding to platelets was barely detectable for both preparations (MFI<5000; **Figure 1A**). However, the comparison between GH-ALG and rabbit ATG binding was questionable because two different detection systems (anti-pig and anti-rabbit antibodies) were used. To accurately compare the binding of GH-ALG and rabbit ATG to platelets, we used AF-488-conjugated protein G to similarly detect both pig- and rabbit-bound IgG. The ability of Protein-G to equivalently recognize pig and rabbit IgG was first confirmed by ELISA and surface plasmon resonance (**Supplemental Figure 1**). The binding of GH-ALG to human platelets was significantly lower, from 100ug/mL to 1000ug/mL, than that of rabbit ATG in the presence of Protein G. Next, we performed platelet assays to determine whether aggregation was altered or induced by GH-ALG or rabbit ATG. No inhibition of induced platelet aggregation was observed with GH-ALG (not shown). Both preparations induced platelet aggregation in this assay but more strongly and faster (visible after 3 or 60 minutes respectively) with rabbit ATG and at lower concentrations (from 250ug/mL) than with GH-ALG (500ug/mL) (**Figure 1C**).

#### Antigenic targets and active fraction

Antigenic targets of GH-ALG were determined using a competition assay against labeled-monoclonal antibodies targeting the classical T-cell markers—*i.e.*, anti-TCR α/β, anti-CD2, anti-CD3, anti-CD4, anti-CD8 and anti-CD28 antibodies. In the presence of GH-ALG, we observed a strong decrease in the MFI for all antibodies evaluated (**Table 1**), indicating competition between mAbs and GH-ALG on these targets. The inhibition of the monoclonal signal ranges from 94% for CD8, 52% for CD4 to 23% inhibition for CD2.

**Table 1:** Antigenic targets.

Differenciation cluster (CD)	MFI		inhibition (1%)
	MFI	inhibition (1%)	
TCR a/b	1304	692	47
CD2	4236	3262	23
CD3	948	591	38
CD4	2005	954	52
CD8	1523	98	94
CD28	535	400	25

Human PBMCs were plated at 1.105 cells per well. The cells were incubated with different labeled monoclonal antibodies (anti-human TCRα/β-FITC, anti-human CD2-PE, anti-human CD3-PE, anti-human CD4-PE, anti-human CD8-PE, anti-human CD28-PE) alone or in combination with GH-ALG at 250 µg/mL. The residual labeling was analyzed by flow cytometry. The results are presented as the mean fluorescence intensity (MFI) and % signal inhibition by GH-ALG.

We then determined the fraction of GH-ALG able to bind target cells. Specific antibodies within GH-ALG polyclonal IgG were removed by successive adsorption on human PBMCs (**Supplemental Figure 2**). The amount of nonspecific GH-ALG antibodies remaining after 5 cycles of adsorption suggested that 40% of GH-ALG IgG was specific to human PBMCs.

### GH-ALG mechanisms of action

ALG/ATG activity classically relies on lymphocyte depletion through complementary mechanisms such as CDC, ADCC, ADCP and apoptosis induction. We have previously shown that GH-ALG is particularly efficient in recruiting human C1q and mediating CDC. By contrast, GH-ALG could not interact with human Fcγ receptors and thus could not trigger ADCC in humans (40). Here, we investigated other mechanisms of lymphocyte depletion using rabbit ATG as a reference to assess GH-ALG activity.

#### Apoptosis

**Figure 2:**
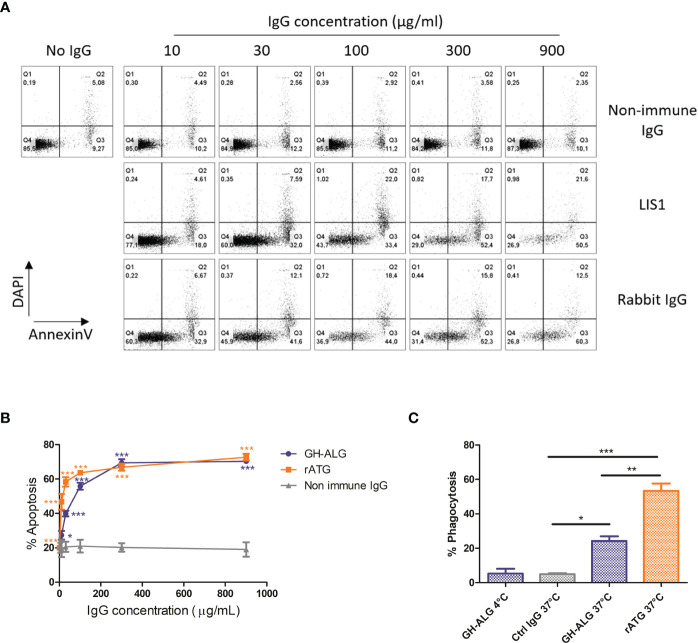
Mechanisms of action of GH-ALG. Induction of apoptosis by GH-ALG. **(A, B)** Human PBMCs were treated with increasing doses (10, 30, 100, 300 and 900 µg/ml) of GH-ALG. Rabbit ATG and nonimmune DKO pig IgG were used as positive and negative controls, respectively. After 3 h of culture (37°C, 5% CO_2_), apoptosis was monitored by flow cytometry by Annexin V and DAPI staining. **(B)** The mean percentage of apoptosis was then determined by adding the percentage of cells in early (Annexin V+/DAPI-) and late (Annexin V+/DAPI+) apoptosis. The data are expressed as means ± SEM (N=3). Two-way ANOVA (*, p<0.5; **, p<0.01; ***, p<0.005), comparison of GH-ALG or rabbit ATG *vs*. nonimmune IgG. Phagocytosis assay. **(C)** Phagocytosis of opsonized PBMCs by human monocyte-derived macrophages was assessed by flow cytometry. CFSE-labeled cells were incubated with IgG in RPMI 10% FCS medium for 30 min at 4°C. Lymphocytes were washed twice and cultured with human macrophages (ratio 1:1) in RPMI 10% FCS. After 3 hours of culture, the cells were washed twice, and macrophages were labeled with CD14-BV421 for 30 min at 4°C. Cells were washed twice before flow cytometry analysis. Phagocytosis was assessed as the percentage of double‐positive (CFSE+/CD14+) cells. The values were compared by ANOVA followed by Tukey’s *post hoc* test. (*, p<0.5; **, p<0.01; ***, p<0.001).

Induction of apoptosis in human lymphocytes was investigated after exposure to increasing doses of GH-ALG. Nonimmune DKO pig IgG was used as a negative control. Induction of apoptosis, including early and late apoptosis, was monitored by flow cytometry using Annexin V and DAPI staining (**Figure 2A**). Significant apoptosis induction was detectable using 10 µg/ml of GH-ALG (**Figure 2B**) and increased with GH-ALG concentration, reaching a plateau at 300 µg/ml.

#### Phagocytosis of opsonized targets

A significant uptake of T lymphocytes by monocyte-derived macrophages was observed after opsonization with GH-ALG or rabbit ATG (**Figure 2C**), with 23% or 53% of macrophages ingesting T cells, respectively. The absence of internalization when cultures were performed at 4°C demonstrated the active process involved. The active GH-ALG concentration was 10 µg/mL, a concentration with limited apoptosis. Therefore, the data presented reflected ADCP activity mediated by opsonization of targets and not phagocytosis of apoptotic cells.

#### Inhibition of T-cell alloreactivity

**Figure 3:**
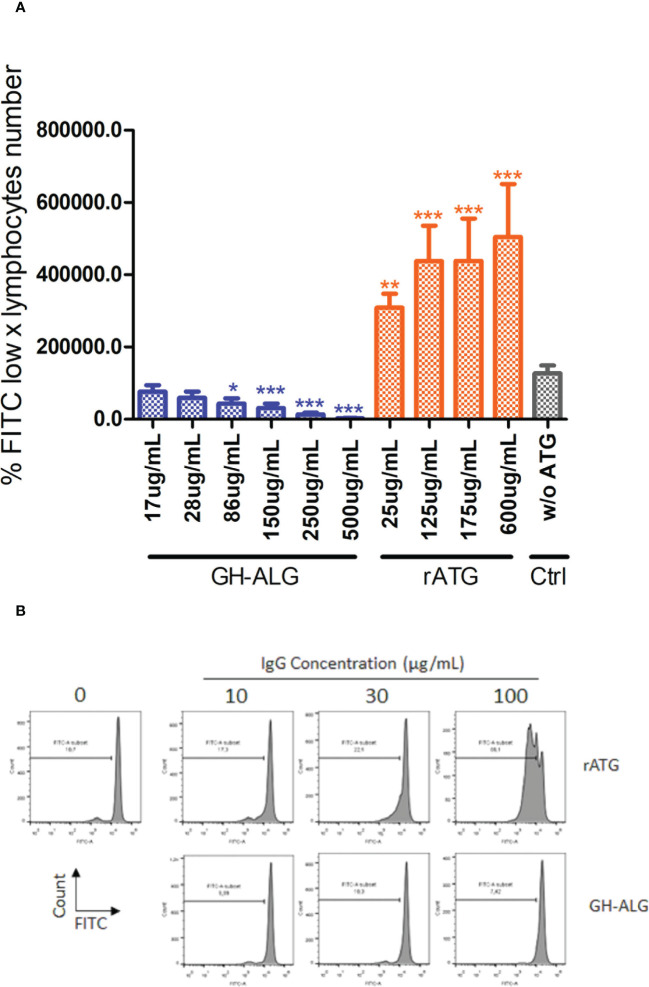
Mixed Lymphocyte Reaction assay. **(A)** Stimulator human PBMCs were irradiated with 35 Gy; responder PBMCs were labeled with CFSE. Stimulator cells were adjusted to 8.106 cells/mL, and responder cells were adjusted to 2 × 106 cells/ml of RPMI medium. Next, the PBMCs were cocultured in a total volume of 200 µl of RPMI medium at 37 °C in a 5% CO2 incubator in the dark for 3 days with or without 250 µg/mL of rabbit ATG or GH-ALG. Proliferation was evaluated by measuring CFSE incorporation using flow cytometry. Groups were compared using ANOVA and the Tukey‒Kramer multiple comparison test. **(B)** Representative flow cytometry histogram demonstrating serial dilution of FITC. * between 0.01 and 0.05. ** between 0.001 and 0.01. *** between 0 and 0.001.

We evaluated the ability of GH-ALG to block T-cell alloreactivity. One-way MLRs were performed in the presence of GH-ALG or rabbit ATG, and T-cell proliferation was monitored after 3 days. We observed a strong and dose-dependent inhibition of residual live T-cell proliferation when MLR was performed in the presence of GH-ALG (**Figure 3**). The inhibition reach 98% at a concentration of 500 µg/ml and remains significant up to 86ug/mL.

### GH-ALG in nonhuman primates

#### Skin graft rejection

**Figure 4:**
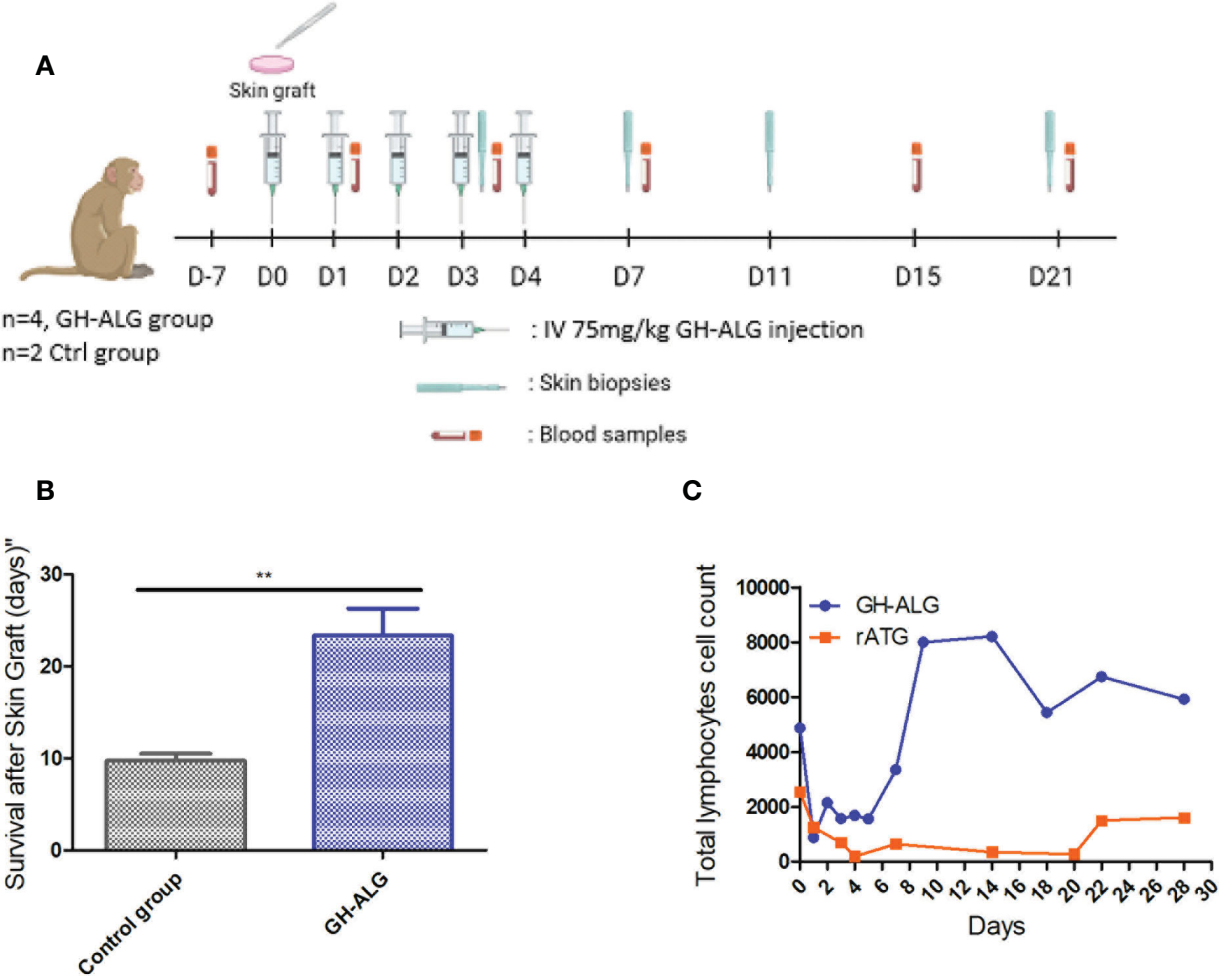
*In vivo* efficacy of anti-lymphocyte IgG in NHP. **(A)** Timeline of the NHP skin graft study. **(B)** Skin grafts were performed on anesthetized animals on Day 0. After back skin shearing and asepsis, the animals were grafted in pairs (back skin collection using a 20 mm diameter template, followed by grafting and skin graft suturing). In the back of each animal, three grafts were performed: one autograft (as a control) and two pairwise allografts (one animal received skin transplants of his congener). Four cynomolgus monkeys intravenously received GH-ALG at 75 mg/kg once a day for 5 consecutive days. Four cynomolgus monkeys were used as controls and did not receive treatment. Skin graft rejection was considered when the area of the necrotized part covered the full graft, and the whole graft site hardened. **(C)** GH-ALG and rabbit ATG-induced T-cell depletion in macaques. Cynomolgus monkeys (n=9) received I.V. daily doses of 50 mg/kg of GH-ALG for 5 days. Cynomolgus monkeys (n=2) received I.V. daily doses of 5 mg/kg of rabbit ATG for 5 days. ** between 0.001 and 0.01.

To determine the ability of GH-ALG to blunt alloreactivity *in vivo*, allogeneic skin grafts were performed in monkeys (**Figure 4A**). Graft rejection was defined by the presence of a scabby aspect, brown coloring with loss of flexibility, or complete necrosis of the epidermis. Rejection of allogeneic skin grafts occurred from day 8 to 11 in the control group (untreated transplanted monkeys). Graft rejection was significantly delayed following treatment with GH-ALG (**Figure 4B**).

#### Total lymphocyte depletion

Depletion experiments were also performed *in vivo* in cynomolgus monkeys. A dramatic drop in the CD3 lymphocyte count was observed after the first injection of GH-ALG or rabbit ATG, from 4.8 giga lymphocytes/L to 0.8 for GH-ALG and from 2.5 to 0.2 for rabbit ATG. (**Figure 4C**). Lymphocyte depletion induced by GH-ALG was limited to one week, the rise above 1giga lymphocytes/L is observed from day 2 post injection.

#### Lymphocyte subpopulations

**Figure 5:**
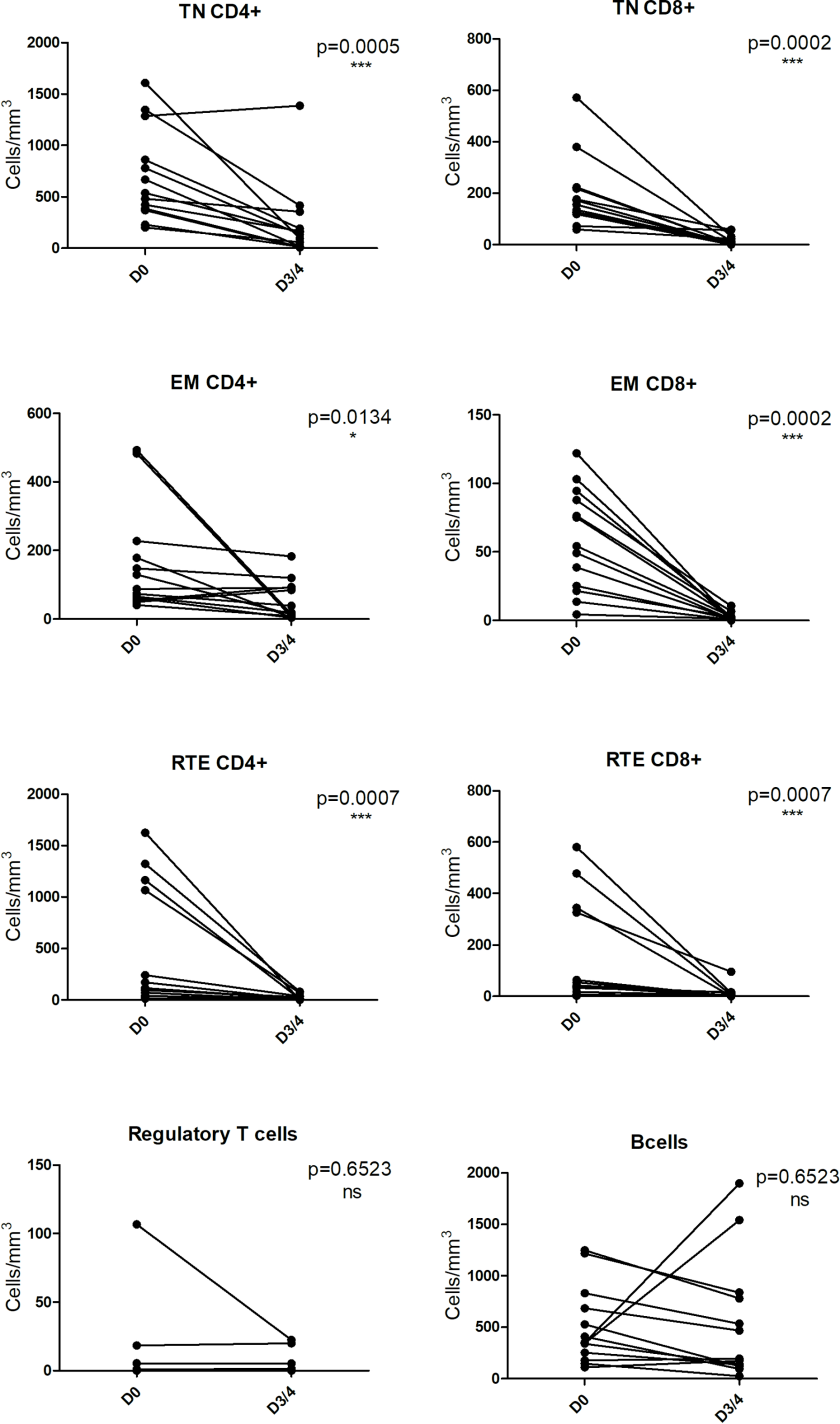
GH-ALG shows selective immunoreactivity to peripheral T cells. Cynomolgus monkeys (n=11) received I.V. daily doses of 50 mg/kg GH-ALG for 5 days. On Days 3-4, blood was sampled, and lymphocyte subpopulations were analyzed by flow cytometry. Whereas CD4+ and CD8+ T-cell subpopulations were reduced, Treg and B cells were unaffected. Monocytes were also unaffected (not shown). * between 0.01 and 0.05. *** between 0 and 0.001.

We investigated the depletion of lymphocyte subpopulations after five I.V. infusions of GH-ALG (50 mg/kg/adm) in cynomolgus monkeys. GH-ALG induced selective depletion of peripheral effector naive T cells (CD4^+^ TN p=0.005; CD8^+^ TN, p=0.0002), memory effector T cells (CD4^+^ EM p=0.0134; CD8^+^ EM p=0.002) and recent thymic embryonic cells (CD4^+^ RTE, p=0.007; CD8^+^ RTE, p<0.007) between Day 0 and Day 3/4 (**Figure 5**). Depletion was stronger for CD8 cells (86% drop for naive CD8 cells and 95% for memory effectors) than CD4 cells (71% decrease for naive CD4 cells and 43% for memory effectors). By contrast, B cells and Tregs were unaffected by GH-ALG (**Figure 5**).

#### Pharmacokinetic study

**Figure 6:**
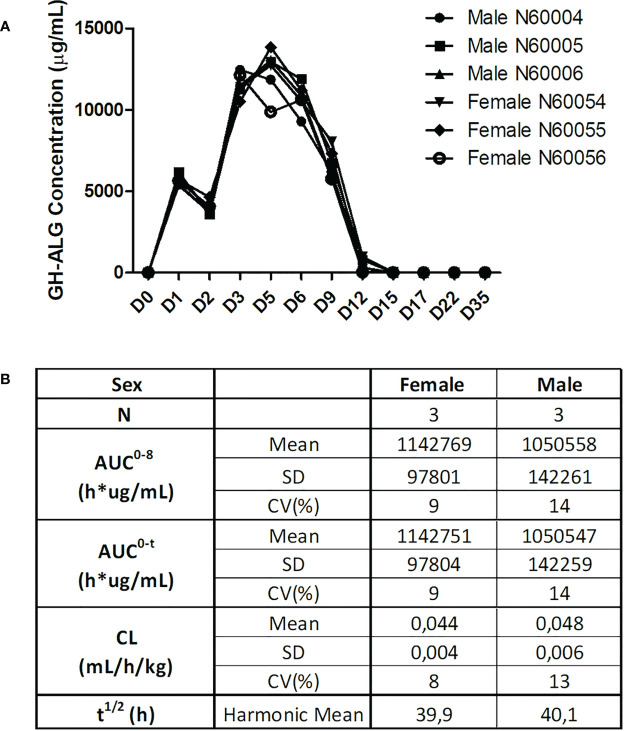
Pharmacokinetic evaluation. For the GH-ALG GLP toxicology study, one group of three male and three female cynomolgus monkeys received GH-ALG by daily intravenous administration (4-hour infusion) at 50 mg/kg/adm for 5 days. Serum samples were drawn on Days 1 (before infusion, 5 minutes, 24 and 96 hours after infusion) and 5 (before infusion and 5 minutes after infusion) and Days 6, 9, 12, 15, 17, and 22 and week 5. Pharmacokinetic parameters were analyzed by ELISA. **(A)** Pharmacokinetic profiles of GH-ALG-treated macaques. **(B)** Pharmacokinetic characteristics.

Serum samples were drawn and analyzed by ELISA. GH-ALG presented a Half-life of approx. 40 h, and a distribution profile compatible with the plasmatic compartment in primates (**Figure 6**). The mean systemic clearance was 0.044 and 0.048 mL/h/kg in females and males, respectively. Quantifiable serum concentrations were observed until the last collected time point (29 days after the last infusion on Day 5), reflecting a slow elimination rate of GH-ALG. No marked difference was observed between males and females, as the ratio of AUC_0-t_ values was close to 1.

## Discussion

GH-ALG is a novel ALS obtained from GGTA1 and CMAH double KO pigs hyperimmunized with a double-positive human CD4^+^/CD8^+^ T-cell line. GH-ALG differs from other ALSs by its mechanism of action, which relies on apoptosis, CDC and inhibition of alloreactivity without ADCC.

The goal of induction therapy is to control early acute rejection following organ transplantation (41, 42). After infusion, rabbit ATG induces immediate immune cell depletion, particularly T lymphocyte depletion, through ADCC, CDC, opsonization resulting in phagocytosis of IgG-coated T cells apoptosis by activation of the Fas-Fas ligand pathway (35, 36, 43). Although these mechanisms allow marked depletion of target cells, they come at the cost of leukopenia and thrombocytopenia. Moreover, in some patients, CD4^+^ lymphopenia lasts for several months, increasing the risk of infection and other complications^41,42^.

One main challenge in organ transplantation is to define the best immunosuppressive strategy to ensure long-term graft and patient survival by preventing graft rejection and protecting against treatment side effects (2, 44, 45). Thrombocytopenia is a frequent complication, partly induced by immunosuppressive therapies. Thrombocytopenia is more common in patients receiving ATGs than in those receiving basiliximab in the first few days after transplantation (2, 45). Although thrombocytopenia is often mild and transient, for kidney transplantation, it may exacerbate the perioperative coagulation imbalance in patients with already compromised bleeding control. Because of differences in the source of cells used for immunization, GH-ALG and other ATGs have different antigenic profiles, and these differences may lead to clinical consequences (46), particularly for thrombocytopenia. We illustrate here that the binding and platelet aggregation of GH-ALG is very low, which might result in lower thrombocytopenia.

The mode of action of GH-ALG includes transient immunosuppression by inducing CDC, ADCP and apoptosis-mediated selective T-cell depletion. Using *in vitro* assays and *in vivo* pharmacodynamic and transplantation models in nonhuman primates (NHPs) or humanized RRGS rats (47), GH-ALG confirmed this mode of action and nevertheless demonstrated a strong impact on T-cell responses in a stringent skin graft rejection model.

Conventionally, ADCP is described as a mechanism by which antibody-opsonized target cells activate FcγRs on the surface of macrophages to induce phagocytosis (48). Thus, the mechanism underlying GH-ALG-generated ADCP is currently unknown because no interaction exists between porcine IgG and human FcγRs, except for FcRn (40). The active GH-ALG concentration was 10 µg/mL, a concentration with limited apoptosis. Therefore, the data presented reflected ADCP activity mediated by opsonization of targets and not phagocytosis of apoptotic cells. Studies have shown that FcRn expression on phagocytic cells (49, 50) increases the phagocytosis capacity of opsonized IgG particles (51, 52). This mechanism of phagocytosis (52, 53) would likely require external sensing and cellular activation *via* FcγR or pattern recognition receptors (C-type lectin receptors) (54). Phagocytosis by neutrophils and monocytes mediated by the interaction between C3b fragment from the cleavage of complement protein C3 and the CR1 receptor (CD35) is also a possible mechanism, although not explored here.

Additionally, *in vitro*, we showed that in mixed lymphocyte reaction (MLR) experiments, LIS1 demonstrated a potent immunosuppressive activity, a feature not found with a preparation of rabbit ATG assessed in parallel. It was well described for rabbit ATG (Thymoglobulin^®^) that at the concentration of 0.1 mg/ml onwards, it activated T cells *in vitro* (both CD4 and CD8 subpopulations) with the synthesis of IL-2, IFN-γ, and CD25 expression, inducing subsequent T cell proliferation (9). Presumably, LIS1 blocks T-cell receptors important for alloreactivity. This feature might be due to the incompatibility between pig IgG Fc domains with human Fc receptors, reducing FcR-mediated cross-linking and agonist events. This mechanism is reminiscent of the mechanism of another class of induction therapy, the anti-CD25 monoclonal antibodies (e.g., basiliximab), which completely and consistently block activated T cells and their proliferation *via* blocking the interleukin-2 receptor.

Rabbit ATG is obtained from rabbits immunized with a preparation of human thymocytes containing mature and immature T cells, Treg cells, progenitor cells, dendritic cells, epithelial cells and endothelial cells. It thus potentially targets a wide range of cells other than T lymphocytes. In a humanized mouse model, rabbit ATG has been shown to bind almost all human hematopoietic cell lineages, including hematopoietic stem cells, and thus likely depletes all subpopulations (55). The prolonged lymphodepletion observed in macaques treated with rabbit ATG (56), might originate from this broad impact of ATG on T cells and stem cells, also impacting T-cell reconstitution. Because GH-ALG is obtained by immunization with a selected effector-type human T-lymphocyte cell line, it shows selective immunoreactivity to peripheral effector T cells but not to B cells, Treg, recent thymic emigrant cells and myeloid-derived suppressor cells described in experimental models and humans to contribute to immunosuppression maintenance post kidney transplantation (57, 58). *In vivo* evaluation in nonhuman primates showed that GH-ALG robustly depleted target T cells from the peripheral blood. Interestingly, depletion was intense but short-term because T-cell counts recovered within two weeks. In a previous study in RRGS humanized rats reconstituted with human PBMCs (47), GH-ALG blocked human T-cell reconstitution by more than 98% and prevented graft versus host disease (GVHD).

Although potent T-cell depletion has been historically considered necessary to prevent acute rejection during the early posttransplant phase for patients with high immunological risk, the prolonged T-cell depletion (35) (59) is associated with an increased risk of infection, including bacterial infections (urinary tract and wound infections) and less commonly viral infections, such as cytomegalovirus (CMV), Epstein−Barr virus (EBV) and BK polyomavirus (BKV) infections (60). Studies have also reported that CD4^+^ T-cell lymphopenia is associated cardiovascular complications and mortality (61). An increased risk of infections is associated with the increased risk of posttransplant morbidity, rehospitalization and overall mortality (62, 63) and with decreased graft survival due to the infection itself and to the reduction in immunosuppressive therapy needed to manage infections (64). Our preclinical data show that after 5 consecutive days of GH-ALG infusion, T-cell depletion occurs acutely over 5 days and returns to baseline levels at the latest within 3 weeks (see **Figure 4**). If confirmed in human, these data suggest that GH-ALG may reduce the risk of infection associated with prolonged depletion observed with other ALS therapies. This regimen would for example benefit patients with a higher risk of infection, among which elderly kidney graft recipients (64). Avoiding prolonged T-cell depletion may also reduce the risk of neoplasm in patients, a possible additional benefit from induction with GH-ALG (65).

GH-ALG presented shorter and milder depleting activity than rabbit ATG and exhibited direct T-cell blockade activity in a mixed lymphocyte reaction. Nevertheless, *in vivo* efficacy data in a stringent skin graft model in cynomolgus monkeys showed an equivalent capacity of GH-ALG to prevent allograft rejection compared to published Thymoglobulin^®^ data (56), 9 to 20-25 days with Thymoglobulin^®^ and 9 to 20-29 days with GH-ALG. Whether the net effect of these differences allows similar protection against allograft rejection still remains to be proven in future clinical studies.

Breg cells prevent graft rejection and belong to the signature of tolerance (66–68). Our study shows that GH-ALG spares B cells, thus avoiding depletion of B-regulators, a major difference from rabbit ATG, which induces apoptosis of all B-cell subsets (6). Similarly, regulatory T cells (Tregs) play a crucial role in limiting renal transplant rejection and, potentially, in promoting transplant tolerance (69). Rabbit ATG induces and expands CD4^+^CD25^+^Foxp3+ cells with regulatory function ex vivo (70). We observed that Tregs were not affected by GH-ALG in nonhuman primates, at least in the short term. Whether these differences in the modulation of regulatory B and T cells may impact the outcomes of induction treatment is unknown.

GH-ALG is currently undergoing clinical evaluation in humans in *de novo* kidney transplant recipients (ClinicalTrials.gov Identifier: NCT04431219). This trial will investigate whether the short-term depletion observed in primates replicates in humans and allows safe induction treatment. The hypothesis of an improved posttransplant outcome due to faster T-cell reconstitution and avoidance of infections, while similarly controlling acute rejection needs to be assessed in larger cohorts.

Our paper suffers from limitations related to the complexity of the mode of action of polyclonal products and of the induction procedure in solid organ transplantation. Ideal duration of lymphodepletion post-induction is unclear, and it is unknown whether the short-term T cell depletion reported with GH-ALG plus the GH-ALG-induced blockade of alloreactivity will be sufficient to blunt acute rejection to the same extent as long-term depleting ATG. Similarly, the second role of induction would be to delay calcineurin inhibitors because of their nephrotoxicity, a shorter depletion may also not be suitable. Although absence of B cell depletion by GH-ALG might preserve Breg cells important in tolerance induction, it is unknown whether this presents advantage or drawback, owing to the possible lower control of anti-donor antibodies. The potentially different active titers of specific antibodies make elusive quantitative comparison of GH-ALG and rabbit ATG. However, our data suggest that GH-ALG may gather a specific pattern of properties that could increase the clinical advantage/disadvantage ratio of polyclonal anti human T cell preparations and widen their clinical usage. We have nevertheless tried to carry out as much as possible our *in vitro* experiments with an equivalent titer. Finally, as many data already exist in the literature for the rabbit-ATG skin grafting model, our grafting model proposes, for ethical reasons, a comparison with control animals and not with animals treated with rabbit-ATG. Our conclusions remain related to the differences in experimental conditions between the studies. As another limitation, we can add that the macaques used in our preclinical model are not CMAH deficient like humans (they are part of the old world monkey classification and have not had a mutation of their CMAH gene) (71), and therefore make Neu5Gc that can be found metabolically on the surface of their cells, but also develop a natural anti-Neu5Ac immunity. As GH-ALG is produced in a DKO CMAH-/GGTA1- pig model, the glycosylations carried by the IgGs are predominantly Neu5Ac. It has been shown (72, 73) that this difference in glycosylation could influence the recognition of PBMCs, which could be a bias to the analysis of our results on lymphocyte populations in the NHP model.

In summary, GH-ALG is a novel induction agent with potential interest for transplanted patients, overcoming problems related to xeno-antigenicity and prolonged target T-cell depletion. Its mechanism of action, combining mild T-cell depletion with inhibition of alloreactivity, warrants its clinical evaluation in kidney graft recipients.

## Data availability statement

The original contributions presented in the study are included in the article/**Supplementary Material**. Further inquiries can be directed to the corresponding author.

## Ethics statement

The animal study was reviewed and approved by the French Research Ministry.

## Author contributions

Conceived the study: J-MB, SB, OD, J-PS, and BV; designed and supervised some experiments: OD, J-PS, and BV; performed the experiments: CC, GE, JR, P-JR, and AS. All authors contributed to the article and approved the submitted version.

## Supplementary material

**Supplemental Table 1** Overview of the in vivo analysis of cynomolgus monkeys. Cynomolgus monkeys (n=16) received I.V. daily doses ranging from 40 to 75 mg/kg GH-ALG for 5 days. The characteristics of these in vivo studies are described here.

**Supplemental Figure 1** Comparison of pig and rabbit IgG detection by G protein: (A) ELISA: GH-ALG and rabbit-ATG are coated on a maxisorp plate at 50ug/mL. After saturation and washing, revelation is performed with Alexa Fluor 488 conjugated protein G diluted at 1/200 or 1/400 and the reading performed in fluorescence with TECAN. (B): Analysis of binding kinetics by BLItz (bio-layer interferometry) of GH-ALG and rabbit-ATG on G protein.

**Supplemental Figure 2** Evaluation of the GH-ALG active fraction. One milliliter of GH-ALG diluted in PBS to a concentration of 1.6 mg/mL was incubated with 30.10^6^ target lymphocyte cells. After 20 min of incubation at room temperature, the supernatant (SN1) was transferred to a second tube containing 30.10^6^ target T lymphocytes. After 20 min of incubation at room temperature, the supernatant (SN2) was collected and stored until SN5. The supernatants obtained were serially diluted and then incubated (30 min, 4°C) with fresh XT1501 cells. After washing, a secondary anti-pig antibody was deposited, revealing the remaining XT1501-specific antibody fraction. GH-ALG was used as a positive specificity control. Nonimmune IgGs were used as a negative control.
