# Supplementary figures and images for: LIS1, a glyco-humanized swine polyclonal anti-lymphocyte globulin, as a novel induction treatment in solid organ transplantation
Source: Front Immunol. 2023 Feb 16;14:1137629. doi: 10.3389/fimmu.2023.1137629 (PMC9978386; doi:10.3389/fimmu.2023.1137629)

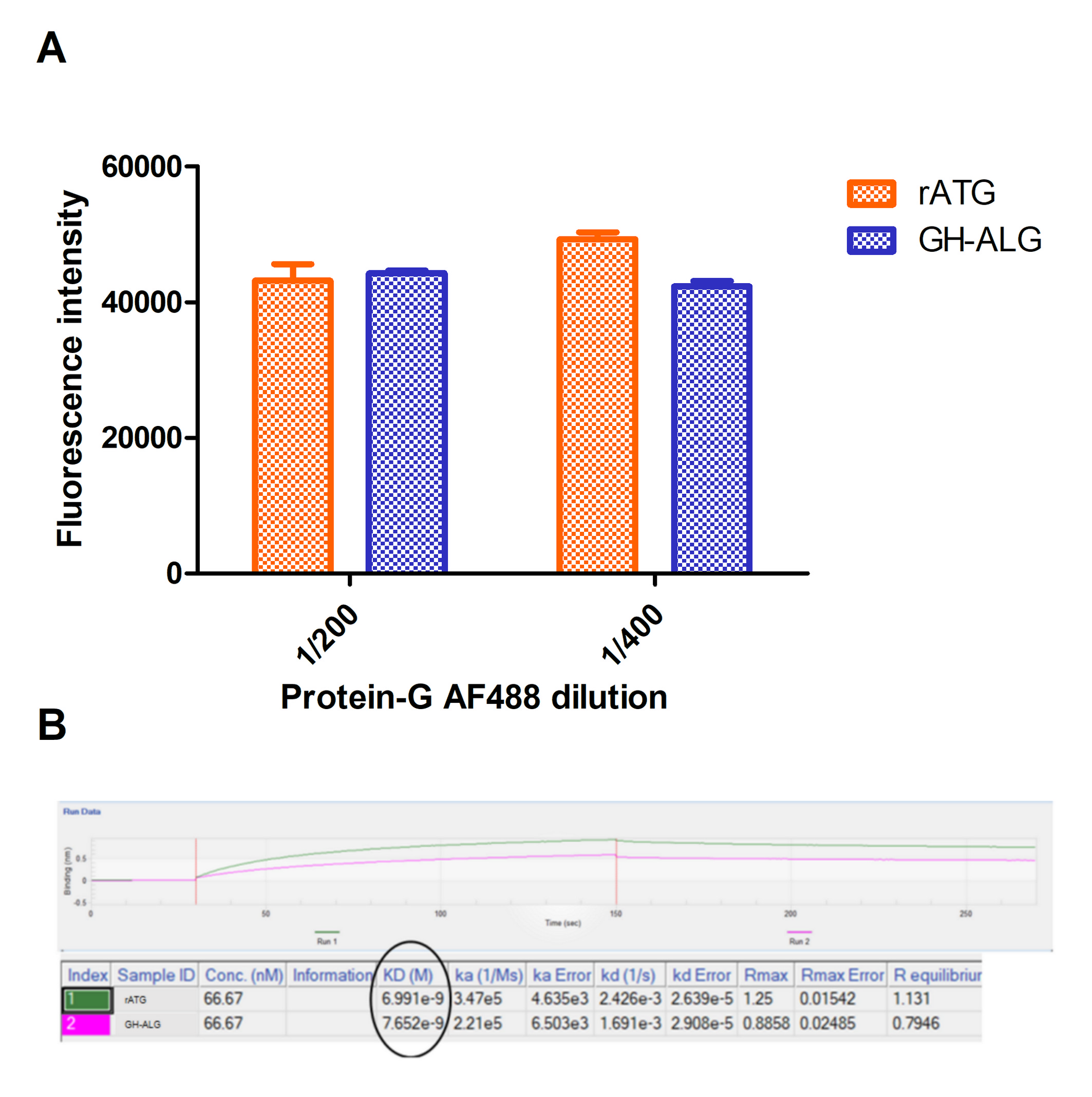

Supplement: Supplementary file 2 — Supplemental Figure 1 Comparison of pig and rabbit IgG detection by G protein: (A) ELISA: GH-ALG and rabbit-ATG are coated on a maxisorp plate at 50ug/mL. After saturation and washing, revelation is performed with Alexa Fluor 488 conjugated protein G diluted at 1/200 or 1/400 and the reading performed in fluorescence with TECAN. (B): Analysis of binding kinetics by BLItz (bio-layer interferometry) of GH-ALG and rabbit-ATG on G protein. [file Image_1.tif]

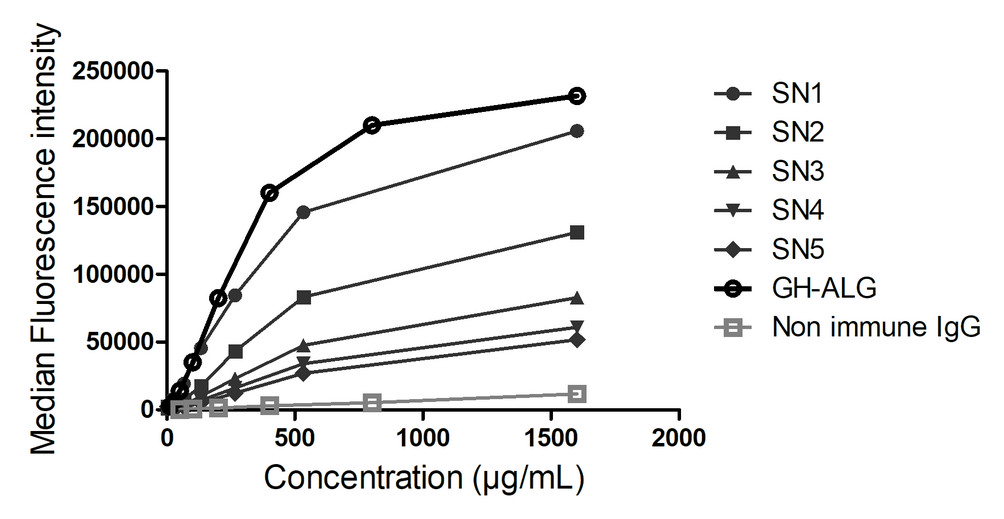

Supplement: Supplementary file 3 — Supplemental Figure 2 Evaluation of the GH-ALG active fraction. One milliliter of GH-ALG diluted in PBS to a concentration of 1.6 mg/mL was incubated with 30.106 target lymphocyte cells. After 20 min of incubation at room temperature, the supernatant (SN1) was transferred to a second tube containing 30.106 target T lymphocytes. After 20 min of incubation at room temperature, the supernatant (SN2) was collected and stored until SN5. The supernatants obtained were serially diluted and then incubated (30 min, 4°C) with fresh XT1501 cells. After washing, a secondary anti-pig antibody was deposited, revealing the remaining XT1501-specific antibody fraction. GH-ALG was used as a positive specificity control. Nonimmune IgGs were used as a negative control. [file Image_2.tif]
